# Quantitative assessment of visual estimation of the infrared indocyanine green imaging of lymph nodes retrieved at sentinel node navigation surgery for gastric cancer

**DOI:** 10.1186/s12893-016-0152-3

**Published:** 2016-06-01

**Authors:** Naoto Takahashi, Hiroshi Nimura, Tetsuji Fujita, Shigeo Yamashita, Norio Mitsumori, Katsuhiko Yanaga

**Affiliations:** Department of Surgery, The Jikei University School of Medicine, 3-25-8 Nishi-Shinbashi, Minato-ku, 1058461 Tokyo Japan; Department of Surgery, The Jikei University Kashiwa Hospital, 163-1 Kashiwa-shita, Kashiwa, 2778567 Chiba Japan

## Abstract

**Background:**

Although the infrared indocyanine green (ICG) imaging is an effective method to identify sentinel lymph nodes (SLNs) of gastric cancer, its objectivity has not been verified.

**Methods:**

We studied 563 lymph nodes under infrared light observation from the ICG-positive lymphatic basins of 36 patients who underwent SLN-navigated gastrectomy for clinically node-negative gastric cancer. First, the rate of SLN detection, the number of SLNs and sensitivities were compared between ordinary light observation and infrared light observation. Second, 563 lymph nodes were grouped into ICG-positive and -negative under infrared light observation. The intensities of the region of interest for each lymph node defined as the lymph node on which digital imaging was performed using an imaging-software, and the region of reference defined as its surrounding background, were compared and quantified.

**Results:**

In the comparison of ordinary light observation with infrared light observation, the SLN identification rates were 28/36 (78 %) vs. 36/36 (100 %), the mean ± SD (minimum to maximum) number of SLNs was 3.4 ± 3.7 (0–16) vs. 9.2 ± 5.9 (2–25), and the sensitivities were 1/5 (20 %) vs. 5/5 (100 %). The ICG-positive group contained 358 lymph nodes with an intensity of 0.323 ± 1.56 (mean ± SD), and the ICG-negative group contained 205 lymph nodes with an intensity of 0.639 ± 1.93 (mean ± SD), demonstrating a significant difference between these two groups (*P* < 0.0001).

**Conclusions:**

The significant difference in the intensity as measured by an imaging-software between ICG-positive and ICG-negative lymph nodes would erase the concern about the objectivity of the infrared ICG method for SLN-navigated surgery for early gastric cancer.

## Background

Sentinel lymph node (SLN)-navigated gastrectomy is a less invasive alternative to conventional radical gastrectomy for early gastric cancer, which has been performed using the tracers in Far East. Tracers currently used for detection of SLNs of gastric cancer are dyes [1, 2] and radioisotopes [3]. Radio-labeled SLNs are defined as hot nodules containing 10 times more radioactivity than surrounding tissue as measured by a gamma probe [3], whereas blue-dyed SLN sampling is based on visual evaluation [1, 2], maybe yielding inconsistency between the observers. We reported that infrared light observation was more feasible than ordinary light observation for SLN-navigated gastrectomy with higher detection rates of SLNs when using indocyanine green (ICG) dye as the tracer [4]. Compared with the radio-guided and combination methods with dual tracers, performance of SLN biopsy with the dye-only method is an inexpensive and practical technique that does not require involvement of the nuclear medicine department. However, some SLNs could be missed with dye-only method because of its nature of visual evaulation [5]. The aim of this study was to quantitatively assess the visual evaluation of the infrared ICG image.

## Patients and Methods

For the analysis, we enrolled 36 patients with appropriate digitization and storage of the removed lymph nodes among those who underwent SLN navigation surgery (SNNS) for early gastric cancer at our hospital. Open gastric resection was performed in 17 patients with a cT1N0 or cT2N0 tumor, and laparoscopic gastric resection was performed in 19 patients with a cT1N0 tumor. We examined a total of 563 lymph nodes retrieved from these patients. All patients provided written informed consent. This study was approved by the Institutional Review Board of the Jikei University Scholl of Medicine (Reference No.15-90) and conducted in accordance with the Good Clinical Practice guidelines and the Declaration of Helsinki.

**Fig. 1:**
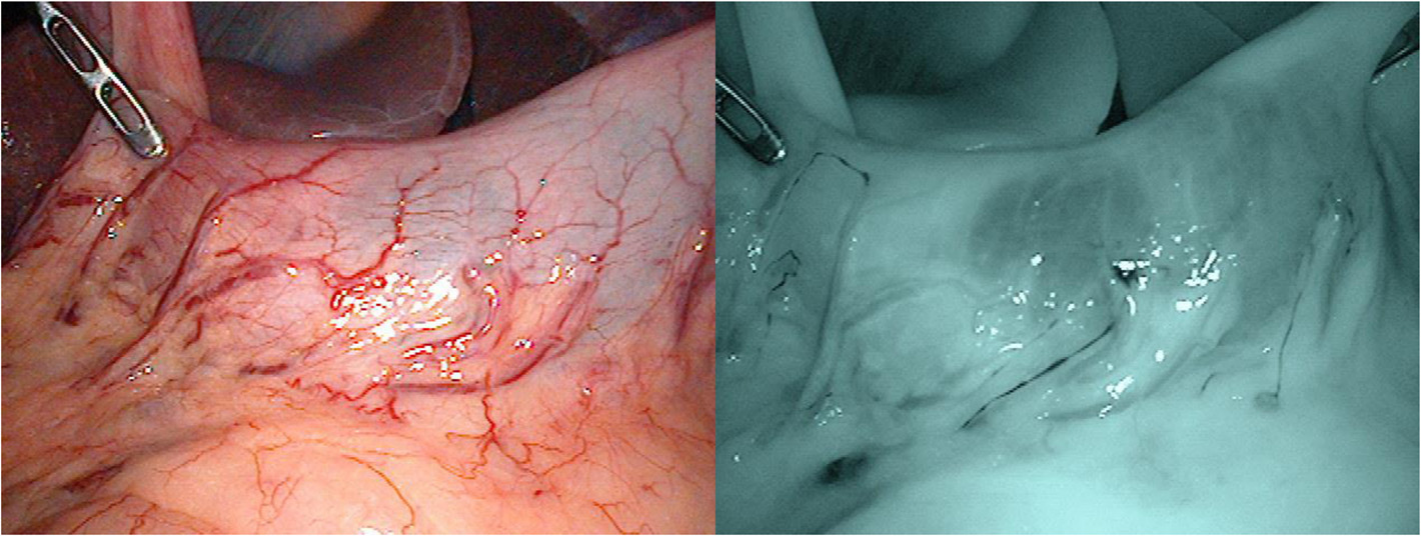
Lymphatic ducts and lymph nodes, which are difficult to see under ordinary light (left), can be clearly visualized under infrared light (right)

**Fig. 2:**
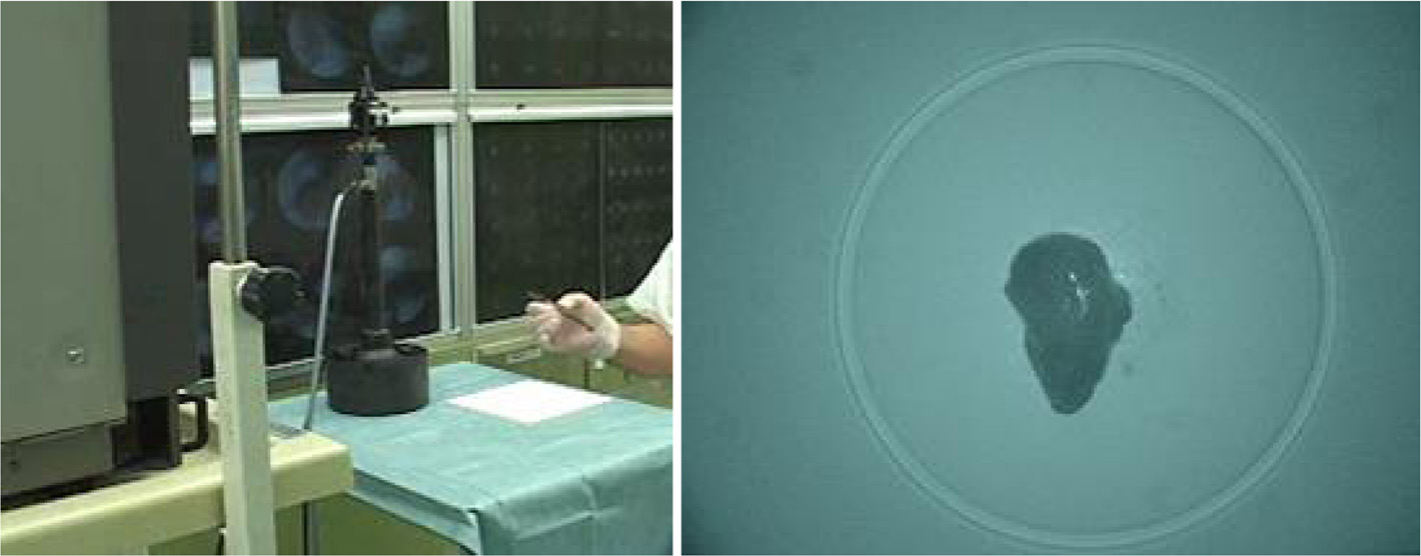
The black box. It is possible to photograph lymph nodes under the same conditions using this black box (left). A picture of lymph node in the black box under infrared light (right)

**Fig. 3:**
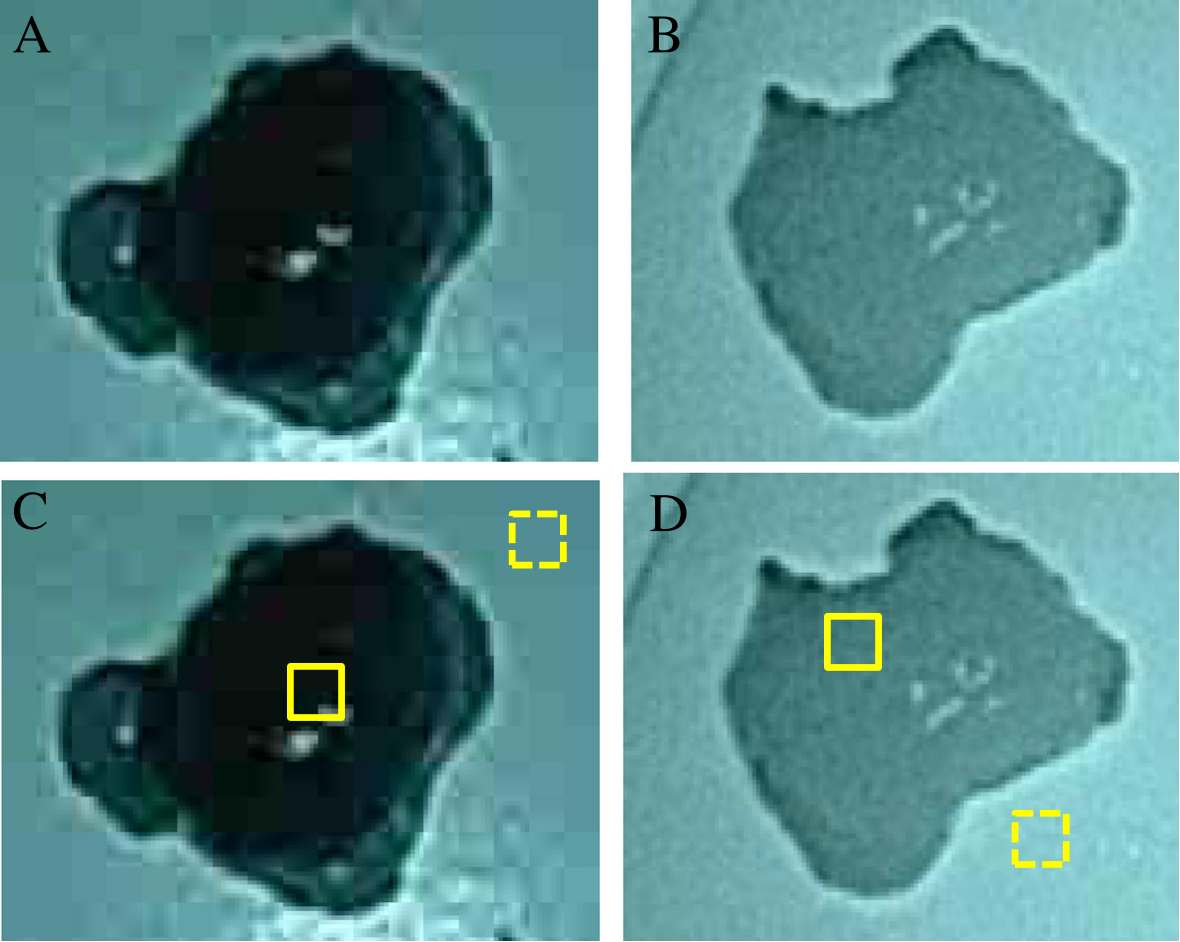
lymph nodes photographed in the black box. ICG- positive and -negative lymph nodes are easy to distinguish **a** and **b**. In the lower line **c** and **d**, the values for the region of reference (yellow dot square) and the region of interest (yellow square) are calculated and quantified using Photoshop, and the ratio of the mean value of the region of interest to the mean value of the region of reference is 0.118 and 0.614, respectively

Zero point five milliliters each of 5 mg/ml ICG (Diagnogreen^®^; Daiichi Sankyo Company, Limited), was injected intraoperatively into four quadrants of the submucosa around the tumor. Then, ordinary light observation followed by infrared light observation was performed by the infrared ray laparoscopic system (IRLS: Olympus Medical Systems Corp.) (Fig. 1). The use of infrared light allows clear observation of ICG-positive lymphatic ducts and the ICG-positive lymph nodes which are barely observable under ordinary light (Fig. 1). The lymphatic basin dissection method [2] was applied for ICG-positive lymph nodes. The lymph nodes in the ICG-positive lymphatic basin were grouped subjectively into ICG-positive and -negative on the back table, and the ICG-positive lymph nodes were determined to be SLNs. Then, images of the lymph nodes were photographed under infrared light observation in the black box, created to avoid the influences of background contrast and ambient light (Fig. 2). Photoshop (Adobe Systems, San Jose, CA, USA) was used as the image-analyzing software. Elliptical marquee tool was set into the interested region of the harvested lymph node and the mean value of the histogram was calculated (Fig. 3). The mean value for the region of reference, defined as the background of the lymph node, was calculated in the same way. The ratio of the mean value of the region of interest to the mean value of the region of reference was calculated. Correlations between the calculated values of ICG intensity and ICG positivity/negativity were examined using the Student’s *t*-test.

## Results

**Fig. 4:**
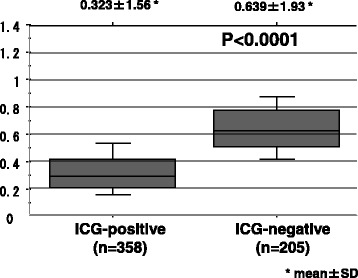
ICG intensity measured in ICG-positive and ICG-negative groups using the Student’s *t*-test was a significant different

In the comparison of ordinary light observation with infrared light observation, identification rates of the SNs were 78 % (28/36) vs 100 % (36/36), the mean ± SD numbers of SNs were 3.4 ± 3.7 (0–16) vs. 9.2 ± 5.9 (2–25) and the sensitivities were 20 % (1/5) vs. 100 % (5/5), showing better outcomes for infrared light observation (Table 1). When the 563 lymph nodes were grouped into ICG -positive and ICG -negative under infrared light observation, 358 were ICG -positive and the other 205 were ICG -negative. The intensities (mean ± SD) were 0.323 ± 1.56 in the ICG-positive group and 0.639 ± 1.93 in the ICG-negative group, demonstrating a significant difference between the two groups (*P <* 0.0001) (Fig. 4).

**Table 1:** Comparison of SNs’ detection by ordinary light and infrared light

	Ordinary light	Infrared ray light
ICG-positive node detection	28/36 (78 %)	36/36
The number of ICG-positive nodes	3.4 ± 3.7^*^ (0 to 16)^+^	9.2 ± 5.9^*^ (2 to 25)^+^
Lymph node metastasis sensitivity	1/5 (20 %)	5/5 (100 %)

^*^mean±SD,^+^(minimum to maximum)

## Discussion

In the advent of SNNS for gastric cancer, Miwa et al. reported that SLNs were detected in 31 of 35 (89 %) patients when they performed SNNSs using patent blue [2]. Kitagawa et al. identified metastatic lymph nodes in 22 of 24 (92 %) patients who underwent SNNSs using the radioisotope method [3]. Upon using ICG as the dye, Hiratsuka et al. reported being able to identify metastases to SNs in 9 of 10 (90 %) patients, but at the same time found metastases to lymph nodes other than SNs in 6 of 9 patients [1]. Ichikura et al. identified metastatic lymph nodes in 11 of 13 (85 %) patients [6]. We revealed that the identification rate and sensitivity of ICG-positive lymph nodes under ordinary light observation were lower than previous reports, while metastatic lymph nodes were identified in all 11 of 11 (100 %) patients by infrared light observation [4]. Moreover; the metastatic lymph nodes were all ICG -positive. The present study also showed an acceptable SLN identification rate, and the sensitivity by the infrared ICG was significantly higher than that of ordinary light observation.

The infrared ray endoscopy system was established as a method for obtaining precise information on the submucosal blood vessels of the stomach, and when used in combination with ICG, which has a maximal absorption near 805 nm, it provides clearer visualization and enables observation to a depth of 3–5 mm [7]. We have reported that infrared light observation was more useful than ordinary light observation in terms of both the SLN identification rate and the detectability of metastatic lymph nodes when ICG was used as the tracer [4]. However, it is possible that some SLNs may be missed when dyes are used for SLN biopsy, because SLN detection is dependent on the ability of the observer to discriminate between ICG -positive and ICG –negative lymph nodes. We therefore attempted to quantitatively assess the visual evaluation of the ICG image. Initially, we tried to store digital images of samples that were mounted on gauze. Resultantly, the background was heterogeneous; photography under the same conditions was not possible. Thus, we created a black box, which enabled lymph nodes to be photographed under the same environmental conditions. A significant difference was found between the values of the ICG-positive and ICG-negative groups by photographing the lymph nodes under the same conditions using this black box and quantifying the digitized images. At presence, it seems difficult to quickly apply the method to clinical practice because of its nature of time-consuming ex vivo examination for quantitative assessment of the infrared ICG images. Radioisotope tracer method is more expensive, cumbersome, and demands specific logistical arrangements due to regulations regarding handling of radioactive material, therefore limiting the number of hospitals which is permitted to use radioactive tracers [8]. Given considerable differences in the intensity (0.323 ± 1.56 vs 0.639 ± 1.93) between ICG-positive and ICG-negative lymph nodes, our study indicates that the current infrared ICG imaging is suitable for SLN biopsy not only in less invasive surgery for gastric cancer, but also for other tumors. In a prospective comparison of the infrared ICG and radioisotope technetium to detect SLNs in breast cancer, among 583 SLNs, 131 nodes (22.3 %) were fluorescent but cold (negative for radioisotope), and 6 (1 %) were not fluorescent (negative for ICG) but hot. Overall, ICG identified more SLNs per patient than did 99mTc (1.94 SLNs per patient vs 1.62 SLNs per patient [9].

## Conclusions

Our quantitative assessment of visual evaluation of the infrared ICG image of lymph nodes retrieved at surgery showed a significant difference in the intensity as measured by an imaging-software between ICG-positive and ICG-negative lymph nodes. These findings would erase the concern about the objectivity of the infrared ICG method.

## Abbreviations

ICG: infrared indocyanine green
IRLS: infrared ray laparoscopic system
SLNs: sentinel lymph nodes
SNNS: sentinel lymph node navigation surgery
